# "The group facilitates everything": meanings patients with type 2 diabetes mellitus assigned to health education groups^1^

**DOI:** 10.1590/0104-1169.0056.2506

**Published:** 2014

**Authors:** Lucas Pereira de Melo, Edemilson Antunes de Campos

**Affiliations:** 2 PhD, Adjunct Professor, Universidade Federal do Rio Grande do Norte, Caicó, RN, Brazil; 3 PhD, Professor, Escola de Enfermagem, Universidade de São Paulo, São Paulo, SP, Brazil

**Keywords:** Public Health Practice, Health Education, Group Processes, Diabetes Mellitus, Type 2, Disease Management, Anthropology, Medical

## Abstract

**OBJECTIVE:**

to interpret the meanings patients with type 2 diabetes mellitus assign to health education groups.

**METHOD:**

ethnographic study conducted with Hyperdia groups of a healthcare unit with 26 informants, with type 2 diabetes mellitus, and having participated in the groups for at least three years. Participant observation, social characterization, discussion groups and semi-structured interviews were used to collect data. Data were analyzed through the thematic coding technique.

**RESULTS:**

four thematic categories emerged: ease of access to the service and healthcare workers; guidance on diabetes; participation in groups and the experience of diabetes; and sharing knowledge and experiences. The most relevant aspect of this study is the social use the informants in relation to the Hyperdia groups under study.

**CONCLUSION:**

the studied groups are agents producing senses and meanings concerning the process of becoming ill and the means of social navigation within the official health system. We expect this study to contribute to the actions of healthcare workers coordinating these groups given the observation of the cultural universe of these individuals seeking professional care in the various public health care services.

## Introduction

Diabetes Mellitus (DM) is a metabolic syndrome resulting from the production, secretion or deficient use of insulin characterized by chronic hyperglycemia, frequently accompanied by dyslipidemia, abnormal blood pressure and endothelial dysfunction. It is considered a chronic condition of a multifactor etiology that requires patients to self-manage their lifestyles. The focus of treatment is to control glycemia, metabolic control, absence of acute and chronic complications, changes of lifestyle and psychosocial adaptation^(^ 1 ^-^ 2 ^)^.

Preventive measures for DM, and its assessment and treatment, include health education actions administered for individuals and/or groups. In Brazil, with the expansion of the Family Health Strategy and the Reorganization Plan of Hypertension and Diabetes Mellitus Care^(^ 3 ^)^, these actions have primarily developed in groups that are coordinated by healthcare workers (physicians, nurses and/or community health agents). In this context, the health education groups directed to diabetic and hypertensive individuals are known as "Hyperdia groups". 

Health education activities should promote opportunities for knowledge and experiences concerning the disease to be shared and exchanged so that patients and their families make conscious and informed decisions about the self-management of their chronic condition^(^ 4 ^)^. There is evidence that appropriate management improves eating habits, decreases levels of blood glucose and glycated hemoglobin, and also decreases the incidence of complications such as retinopathy and nephropathy^(^ 5 ^)^.

Given the previous discussion, the question raised in this study is how do individuals affected by DM signify the experience of participating in Hyperdia groups? In these terms, we note the need for an approach that is sensitive to the contexts in which the different healthcare workers and patients "meet". Therefore, in addition to the most general aspects of everyday life of individuals with DM, we should also pay attention to their representations and experiences in the places where they receive care, as well as the political and economic factors that inform the nature and content of healthcare delivery^(^ 6 ^)^.

The objective was to interpret meanings type 2 DM patients assigned to health education groups. Studies of this nature enable the understanding of dimensions concerning the experience with a chronic disease, as well as the interactions of the individual and his/her support network with the official healthcare system, contributing to the delivery of nursing care that values and mediates the knowledge and practices of both popular and erudite models of the disease. 

## Method

Medical anthropology was the theoretical-methodological framework used in this study; specifically, we linked interpretative anthropology^(^ 7 ^)^ and critical medical anthropology^(^ 8 ^)^. This strategy was intended to associate the interpretation of symbolic aspects and cultural meanings assigned to a social phenomenon (typical of interpretive anthropology) with a macro-social perspective from analyses in critical medical anthropology with its focus on the ideological, political, economic, and historical dimensions. Therefore, culture was seen as a material and symbolic system, which, through signs, symbols, codes, cosmologies, values and standards, offered a matrix of meanings within which individuals interpret the world, produce meanings and guide their knowledge, practices and experiences in a given context. Additionally, this matrix of meanings is constantly produced and updated by the action and creativity of social subjects. 

The methodological framework used was ethnography because of its explanatory nature. Ethnography is acknowledged as a theory of practice that comprises social life as a result of interaction between the structure and agency of individuals in their daily practices in a given sociocultural context^(^ 9 ^)^. From an operational point of view, ethnography involves a continuous effort to put the researcher in specific meetings and events in order to understand contexts and phenomena significant to a given social group.

This study's fieldwork took place between August 2011 and September 2012 in a healthcare center in the North Health District in Campinas, State of São Paulo, Brazil. During this period we attended Hyperdia groups' meetings, accompanied visitations to patients at home (medical consultations and reception consultations), team meetings and other events pertinent to the study. The informants were selected from five Hyperdia groups conducted by an Extended Family Health Team in this healthcare unit that were created in 2001. In total, there were 113 patients enrolled in the groups and the average duration of participation was five years.

The healthcare team worked with the groups every week and the groups were distributed in such as way that each group met every 45 days on average. The number of the participants per meeting was around 15 patients, in addition to one physician, a nursing auxiliary, and one community health agent. The meetings were held on the healthcare unit premises on Mondays from 1pm to 3pm. During the meetings, the patients had their capillary blood glucose, weight and blood pressure checked. The lectures addressed themes related to DM management and, at the end, the physician individually assessed each patient. In these consultations, the physician provided medical prescriptions, performed and assessed laboratory and image exams, assessed clinical parameters and checked care actions provided in other healthcare services (referral and counter-referral). 

The study was conducted with 26 informants (patients). The sample was intentional. The patients were personally invited to participate in the study during meetings of the Hyperdia groups. The inclusion criteria were: having a DM diagnosis and having participated in the Hyperdia groups of that healthcare unit for at least three years.

Four procedures were used to collect data: participant observation, social characterization, discussion groups (DG), and semi-structured individual interviews. Social characterization was collected through a structured questionnaire. Four DG were performed, each lasting an average of 60 minutes. A total of 18 informants participated: four individuals participated in DG I; six in DG II; three in DG III; and five in DG IV. The interviews aimed to deepen information that emerged in social situations and in the DGs. A total of eight interviews were conducted. The interviews were held by a single researcher and lasted 40 minutes on average. The semi-structured script with guiding topics was previously tested and used in DGs and individual interviews were performed afterwards.

The DGs were held within the healthcare unit premises, while the interviews were conducted either in the patients' homes, workplaces or the healthcare unit. Data were audio-recorded with the consent of the participants and transcribed and coded immediately after the interviews. Information collected during participant observation was recorded in a field diary.

Data analysis occurred in four steps and was concomitant with the data collection. Each case was briefly described in the first step: information regarding the interviewees; the context in which the interview was held; and identification of main topics. In the second step, the first case was deepened and data were codified based on the study's theme, objectives and theoretical assumptions. In the third step, the remaining cases were analyzed and meanings-units were identified through grouping similar and different codes. That is, the first phase of interpretation took place at this point, from which units of meanings emerged. In the fourth and final step, excerpts were analyzed in greater detail. The entire *corpus* was analyzed and a table was created where the core meanings were defined. Finally, the thematic categories were created based on the core meanings^(^ 10 ^)^.

The project was approved by the Institutional Review Board at the University of São Paulo, College of Nursing. The informants signed free and informed consent forms. The patients were identified by randomly chosen fictitious names.

## Results

The social characterization of the participants was: 75% were women aged 67 years old on average; 29.8% were born in the state of São Paulo, showing a significant number of immigrants; 46.2% reported mixed race, and 38.5% reported being Caucasian. In regard to religion, 71.2% reported being Catholic. Despite some widowed participants (23.1%), most were married and lived with their spouses (65.4%). Of the total of participants, 23.1% reported no schooling and 59.6% had from 1 to 4 years of schooling. Family income ranged from 1 to 2 times the minimum wage (32.7%) and from 2 to 5 times the minimum wage (53.8%). The households were mostly characterized by enlarged or extended families: other relatives were included in the family, especially grandchildren, sons- and daughters-in-law. In regard to occupation, 30.8% were currently working or had worked, most of their lives, as domestic workers, followed by 17.3% who worked in agriculture, countryside, farm or fishing, and 19.2% were unpaid homemakers.

After analysis and interpretation of the empirical material, four thematic categories emerged: (1) ease access the service and healthcare workers; (2) guidance regarding diabetes; (3) participation in groups and experience with diabetes; and (4) sharing knowledge and experiences. The emic content of each is presented below.

### Ease access the service and healthcare workers 

The informants considered that participating in the Hyperdia groups was a way to ensure easy access to healthcare services. It meant the possibility of circumventing a bureaucracy existing in the facility and healthcare system that is still characterized by lines, taking a number in a line, appointments, and forms. The participants highlighted the ease in scheduling individual appointments with the physician. *If it wasn't for this group, we would have to come to the facility and schedule an appointment, wait our turn. So, I think the group is medicine for sick people! The group facilitates everything, we don't need to get in line, nor wait for anything. The groups are already scheduled and happen almost every month (Jael). *Another aspect of ease arises from it, as well: no need to get in line for consultations, that is, they did not need to wait the average time between scheduling a consultation and the consultation.

They also emphasized the "privileges" afforded to patients who attended the groups' meetings regularly. They would not need always to get tokens in the reception of the healthcare facility because the professionals already knew them: *Sometimes I get there [to reception], and even if it's not a group day, I show my card (hyperdia card) and go there with the staff and have my consultation immediately. Sometimes I don't even need to get a number (token in the reception). I already know [professionals and place]. I go and they provide consultations right away (Renan)!* Additionally, when needed, they were given priority referrals to other healthcare services: *If I'm not well, the physician gives a referral letter and it's easier to get service. It can take a long time in the healthcare unit, sometimes. The group is good because of it, that it won't take long (Tony). The patients could be scheduled with a physician more easily: It's good because sometimes you don't have time to go there [to the medical consultation] to get a prescription. So, I have this appointment in the group and it helps me a lot (Luiza). *It is important to note that such aspects of ease and "privileges" were obtained through maintaining the bond with healthcare workers and, particularly, with the physician.

### Guidance regarding diabetes 

From the informants' points of view, receiving orientations helped them to know the disease better, which does not necessarily imply modified lifestyles; and especially "seeing" their health status through "controlling" glycemia, weight, blood pressure. *They monitor you every month, see, measure, check how you're doing (Fara). You have to come because, suddenly I may not know how my diabetes is and it goes up all at once! I have to control it the best I can. I come here to know something, get some news, see how we're doing (Lea).*

In this sense, "know something" means to "see" the parameters used and that the professionals give priority to. Hence, they could "control" parameters, i.e., monitor these parameters so they would not be taken by surprise. *It's important to attend the group because your diabetes may be high, you feel the symptoms but you don't know what it is. Here in the group you go through a medical consultation. Your appointment is today and you see right away. We have benefits (Paula).*

### Participation in the groups and experience of diabetes 

The impact of participating in the Hyperdia groups was expressed by the patients through a complex system of opposition contained in two categories formed by structural pairs. The first pair: improvement x control. The emic category "improve" corresponded to the notion of control in biomedical language and referred to the patients' efforts and that of their social support networks to maintain glycemic levels and other physiological and biochemical indicators within the parameters recommended by biomedicine. *It got better, but sometimes it gets high, then it gets low. Sometimes it's too high. It improved a lot! I was much more obese. I had 110 kg. Now I have 93 kg (Daniela). Ah, it improved a lot! Because things we didn't know were clarified. If you're there, they examine [capillary glycemia], measure your blood pressure, and then tell you: 'look, your blood pressure is high!' I was taking Captopril, and every time my blood pressure was a little higher, for this reason she [physician] changed it. Everything is much easier there. For me, the group is a blessing (Fara).*

The second pair: concern x tranquility, emerged from the relationships between patients and healthcare workers, especially the doctor-patient relationship. The informants' reports showed concern for those who did not "seek" the service, that is, those who did not access the official health system. The feeling of tranquility somehow reflected the deficiencies of empowerment of social subjects. *When I started attending the group I became less concerned with my blood pressure, with the diabetes, with other things, you know! So, we already are in the group and she [physician] would talk and make referrals. Before I started the group I had to wait for the day and time [of consultation] (Rui). After I started attending the group, I became more tranquil. I'm not concerned about making appointments: 'Do you want it? Go there' (Luiza).*

### Sharing knowledge and experiences 

Sharing knowledge and experiences concerning DM was a recurrent theme among the informants during the groups' meetings. *That's what I'm saying: you always learn something! You leave there and take it. Because each one has a story to tell! It's good. They exchange ideas, they're from the neighborhood you know, so everybody knows each other and I didn't. I met them in the group (Luiza).*

Nonetheless, most of these exchanges were restricted to conversations among small groups before the meeting started. Even though everyone (workers and patients) was aware of these "whispers" (side conversations), they tended to see these conversations as secondary due to the centrality of the "talks" (professionals' speeches). Hence, the informants reported there was little time to talk in the meetings. *There is a lot of little exchanges, but it's difficult. You can't talk much because if you let them, one tells one story, then someone tells another, and suddenly, everybody is talking. But the time you have [the duration of meetings] is not long. We don't stay there for much time. They have to give you a message ["speeches"]; they transmit something (Carlos).*

Some also emphasized the learning and exchanges, as well as the support received, the friendly words, encouragement to carry on, fun, and relaxation during conversations, the effects this environment had on their "minds" and how it all improved how they cope with DM, with sorrow, and loneliness. *For me it's the best thing I've done. My diabetes and depression improved a lot. Before I started attending the groups, I'd stay home by myself, would spend the entire day crying. Here, I talk, I know everybody! (Daniela) We come, get together with one group and talk, tell stories and it's fun. Because I stay home alone in the afternoon, when my children go to work. Here, I talk, I see people around, it makes me happy! But when I see myself alone, tears fall down! (Ana) Sometimes a word: 'ah, they told me to take this medication that is good for diabetes!' 'Ah, you know what they told me?' 'You want to do this and that, eat this and that, but it worsens your diabetes!' [laughs] So, we support each other! (Neide)*

## Discussion

The most relevant aspect of this study's results refers to the social uses that the informants conferred to the Hyperdia groups. Through an analysis that valued the specificities of these data, we opted to work with basic social elements and mechanisms that characterize everyday Brazilian life with its rituals and models of action, as the following discussion shows.

From the perspective of health policy, the Hyperdia groups are therapeutic devices that implement actions of health education, regular clinical follow-up, periodical control actions, supply medication, and provide care in the event of complications. Such actions should be directed by the individual - an autonomous, responsible, rational and conscious subject^(11) ^- and take place in healthcare facilities that tend to be generalizers, universal, bureaucratic and hierarchical services. In summary, health policy adopts a notion of an atomized individual, separated from a broader social context, and notably separated from relationships. There is little or no consideration for space and time for relationships take place among people, something that is central in the rituals and models of action of Brazilian people^(^ 12 ^-^ 13 ^)^.

Ethnographic data, however, show general aspects concerning the action and creativity of the individuals, the target of this policy, with a view to address this dilemma between the individual and person. Therefore, from this confrontation between the individual's role (universal laws) and the person's role (relationships) emerge coping strategies (trickery, a knack for the system, "do you know whom you're talking to?") through which Brazilians manage to discover and find a way, a manner, a style of social navigation that passes between the lines of these confrontations^(^ 13 ^)^.

In this sense, facility in accessing services and healthcare workers as reported by the informants reveals the social uses of the Hyperdia groups as the patients used trickery to circumvent difficulties of access imposed by bureaucracy and deficient structure and working conditions. Therefore, the groups, instead of presenting opportunities to learn and encourage treatment adherence, were used by the patients to produce, maintain and use networks of relationships that enabled them to "navigate" within the official health system.

**Figure 1 -:**
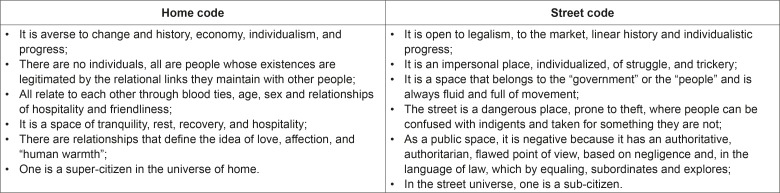
Characteristics of the social universes: stress and home adapted from DaMatta(12).

In addition to this distinction between the individual and the person, it is worth noting the basic social mechanism, within everyday Brazilian life with its rituals and models of action, through which a strong and permanent relationship is established among three spaces and dramatic plans as a way to remake the unit of society: home, street and the other world, as shown in Figure 1*. These are spaces of social signification in which different and complementary social codes are present and normalize and moralize the subjects' behaviors^(^ 12 ^)^.

From this analytical perspective, the health system reflects general aspects of the broader Brazilian society (stratifications, hierarchies, bureaucracies, etc.). The street code and the condition of the atomized patient operate in the healthcare services. In this context, facilitating access meant to personalize the condition of being a patient and recover the home code. The informants did it through their network of relationships, the core element of which was the bonds or "friendship" established with the healthcare workers, notably with the physician.

The means of social navigation constructed by the informants also mean the possibility of using the official health service network as a citizen's right; before the SUS (Unified Brazilian Health System) and Family Health Strategy were implemented, this population was underserved by the nation's health policy.

Under these circumstances, the bond and empathy established with the professionals in the impersonal universe of the health service (street code) recovered the relational and personalized dimensions that are inherent to the home's social universe. It was essential, because diabetic patients reported that the empathy of healthcare workers as manifested by an understanding attitude, attentive listening, and holistic approach, produced a feeling of trust and motivated them to become more involved in the management of their disease^(^ 14 ^)^.

In regard to the guidance and information provided regarding DM, this study's results show the meanings patients assigned to these orientations and the recommendations contained in the biomedical discourse relativizing them. They interpreted the information provided by the healthcare workers and found their own solutions to the particularities of each one's life. These interpretations were notorious for the use of terms such as "measure", "see", and "know". The participants used these terms as corresponding to the technique of the exam in the physician's vernacular. In biomedicine, exams play the role of inverting the "invisibility" of DM (usually asymptomatic) and bringing forth elements the patient could hide. Thus, the exam transforms the individual into a describable object, under the control of permanent knowledge, and enables the exposure of singular traits, his/her particular evolution, and inherent skills or abilities^(^ 15 ^)^.

In contrast, for the informants, "measure", "see", and "know" enabled the production of coping strategies for the irregular capillary glycemia, food exaggerations, and drug therapy. The anthropological literature specializing in health has shown that what reminds these individuals of DM is the daily monitoring of blood glucose, time of medications, dietary restrictions, the need to perform physical activities, and when necessary, insulin injections^(^ 16 ^)^.

Therefore, the expression "*know somethin*g" is consistent with daily efforts to keep clinical parameters "under control" and not being "taken by surprise". This study's data corroborate the existence of two conceptions of "control" already reported in the literature: a biomedical conception, which means keeping glycemia and other parameters within normal values; and a popular conception, which refers to practical concerns that mobilize patients to promote adjustments in their prescriptions, trying to balance them amidst non-medical demands (family, work, religion) that need to be managed in life^(^ 17 ^)^. These same elements are perceptible in the structural pair "improvement x control".

The structural pair "concern x tranquility" shows the demands of patients for medicalization, considering both productive effects and negative aspects. In the face of "concerns" arising from their everyday lives, the informants count on the effects of "tranquility" promoted by the tutelary relationships of care shared with the healthcare workers. Therefore, as the patients projected on the professionals the responsibility for making decisions regarding their care, they softened their accountability for self-care as it is posed by the moral imperative existing in the health policy.

Conversely, in other contexts, involvement in decision-making regarding the disease is seen as essential for patients; healthcare workers are expected to have the competence to work together with patients and recognize and value their knowledge and experiences with DM^(^ 14 ^)^.

Finally, we highlight that knowledge and experiences are shared among the participants during the meetings in the Hyperdia groups. Even though the groups are mainly focused on the development of educational actions and other activities linked to the clinical management of disease, the informants noted the importance of these interactions to helping them cope with DM.

It is worth noting that sharing knowledge and experiences enable a number of actions: identification with other people experiencing similar situations so that individual problems become common problems; alleviation of loneliness and social isolation; relational conditions to put life into perspective; and improving self-perception and family relationships and relationships with healthcare workers. Additionally, identification with others' experiences generates learning and the development of coping strategies and adaptation to daily variations in life^(^ 18 ^-^ 19 ^)^. These aspects in the studied groups need to be known and valued by healthcare workers.

## Final Considerations

This study sought to interpret the meanings diabetic patients assign to the Hyperdia groups. The "anthropological lens" enabled the identification of the informants' social uses of the Hyperdia groups, as well as other aspects related to the experience of DM. Therefore, the studied groups showed themselves to produce instances of senses and meanings concerning the process of becoming ill and the means of social navigation used within the official health system. In this process, ethnography contributed by providing the inter-subjective experience that takes place in the field, its craft nature, and situations of otherness that emerged.

The study enabled grasping the means of social navigation constructed by the informants as a way to circumvent the bureaucratic, impersonal, and hierarchical nature typical of healthcare services. The use of Hyperdia groups to facilitate access to primary healthcare revealed the attempts to introduce the logic of interpersonal relationships, interpretations, the production of meanings, of the symbolic, the completeness of being and its needs, in these contexts. At the same time, the Hyperdia groups exposed the weights and scales that enable interference in personal relationships with the universal law imposed by health policy. These abnormalities that are produced are manifested in the "privileges" conferred onto the groups' participants.

Additionally, we presented the meanings assigned to the orientations regarding DM and the information on it that were transmitted during the groups' meetings. Such meanings express the interpretations of the informants concerning medical speech and the naturalized terms such as "control" and "exam". This study's informants view the groups as spaces to share knowledge and experiences. Even though these exchanges were confined to small groups in side conversations when the professionals had not yet arrived (before the meetings started), they influenced the daily coping with the chronic disease.

Finally, the meanings discussed here concerning the Hyperdia groups may serve as a basis for the actions of healthcare workers coordinating these groups based on the observation of the cultural universe of these individuals seeking professional care in the various therapeutic devices available within SUS. 
